# Adherence to plant-based dietary patterns in relation to glioma: a case–control study

**DOI:** 10.1038/s41598-021-01212-7

**Published:** 2021-11-08

**Authors:** Seyed Mohammad Mousavi, Mehdi Shayanfar, Somaye Rigi, Minoo Mohammad-Shirazi, Giuve Sharifi, Ahmad Esmaillzadeh

**Affiliations:** 1 Department of Community Nutrition, School of Nutritional Sciences and Dietetics, Tehran University of Medical Sciences, P. O. Box 14155-6117, Tehran, Iran; 2 Department of Clinical Nutrition and Dietetics, National Nutrition and Food Technology Research Institute, Shahid Beheshti University of Medical Sciences, Tehran, Iran; 3 Department of Neurosurgery, Loghman Hakim Hospital, Shahid Beheshti University of Medical Sciences, Tehran, Iran; 4 Obesity and Eating Habits Research Center, Endocrinology and Metabolism Molecular Cellular Sciences Institute, Tehran University of Medical Sciences, Tehran, Iran; 5 Food Security Research Center, Department of Community Nutrition, Isfahan University of Medical Sciences, Isfahan, Iran

**Keywords:** Cancer, Neuroscience

## Abstract

Available evidence suggests a favorable association between adherence to a plant-based diet and disease prevention, but data on the link between such dietary intakes and cancer are scarce. We examined the association between the overall plant-based diet (PDI), healthy plant-based diet (hPDI), and unhealthy plant-based diet (uPDI) and risk of glioma. This case–control study was conducted on 128 newly diagnosed glioma patients, and 256 hospital-based controls. Cases were diagnosed by pathological test and controls were selected from hospitalized people in orthopedic and surgical wards. Dietary intakes were assessed using a validated Block-format 123-items food frequency questionnaire. Scores of plant-based dietary patterns were calculated using the method suggested by Satija et al. After controlling for potential confounders, individuals with higher scores of PDI (OR: 0.54, 95% CI: 0.32–0.91, *P*-trend < 0.001) and hPDI (OR: 0.32, 95% CI: 0.18–0.57, *P*-trend < 0.001) had significantly lower odds of glioma compared with those with the lowest scores. This association did not change in the fully adjusted model; such that subjects in the highest tertile of PDI and hPDI were 69% and 71% less likely to have glioma compared with those in the lowest tertile. In contrast, higher scores of uPDI was significantly associated with a greater odds of glioma (OR: 2.85, 95% CI: 1.26–6.47, *P*-trend = 0.02). Adherence to PDI and hPDI was associated with a lower odds of glioma, while greater adherence to uPDI was directly associated with the likelihood of glioma. Further prospective cohort studies are needed to examine our findings.

## Introduction

Glioma is the most frequent and aggressive type of brain tumors; Almost 30% of all brain tumors and 80% of all malignant tumors have been reported to be the cancer of glial cells^1,2^. The global incidence rate of this cancer is 3.7 per 100,000 for males and 2.6 per 100,000 for females^3^. In Iran, the latest data estimated that the mortality rate from brain tumors was 2.92 per 100,000 in men and 2.46 per 100,000 in women^4^. Despite the low incidence rate, the main concern is its high mortality rate; more than 97% of subjects affected with glioblastoma die within 5 years after diagnosis^5^. This highlights the need for recognizing determining elements in the occurrence and development of abnormal proliferation and transformation of non-neuronal cells^6^.

Although several epidemiological investigations have reported the role of diet as a modifiable factor in glioma pathogenesis, most have focused on individual food items, nutrients or food groups^7–10^, and limited documents have considered the joint impact of dietary ingredients through applying dietary patterns. In light of the fact that dietary components are consumed in the context of mixed meals with their probable additive or synergistic effects on each other^11^, assessment of the role of dietary patterns in the pathogenesis of health outcomes is a priority. In this regard, greater adherence to the low carbohydrate diet^12^, Dietary Approaches to Stop Hypertension (DASH)-style pattern^13^, and Mediterranean diet^14^ were inversely associated with the odds of glioma. In contrast, a positive relationship was reported between dietary inflammatory index and risk of glioma in earlier studies^15^. Plant-based diets, which encompass a diverse family of eating patterns, include plant foods such as legumes, whole grains, vegetables, fruits, nuts, seeds, and lack some or all animal foods in their context^16^. Plant-based diets emphasize on the quality of plant foods, which is lacking in the previous healthy dietary patterns^17^. In recent years, great attention has been drawn to the detrimental association of several plant-origin foods, such as refined grains, potatoes, and sugar-sweetened beverages on health outcomes^10,18,19^. A growing body of literature indicates that healthy and unhealthy plant-based diets can affect health outcomes differently in terms of type. For instance, healthy plant-based diets with a greater proportion of nutritive plant foods and a lower amount of animal foods, and less nutrient-dense plant foods (high in refined carbohydrate) was inversely associated with type 2 diabetes and coronary heart disease. In contrast, unhealthy plant-based diets with a high quantity of less healthful plant foods were associated with a greater risk of these conditions^17,20^. Earlier studies have also reported favorable associations between adherence to healthy plant-based diets and risk of breast cancer^21^, non-alcoholic fatty liver disease^22^, and psychological disorder^23^. During glioma progression, reactive oxygen species (ROS) are activated and resulting in oxidative stress (OS)^24^. To avoid oxidative stress, these ROS can be inactivated by antioxidants like glutathione, vitamin E, beta carotene, and vitamin C^25^. Plant-based diets are high in antioxidants and anti-inflammatory phytochemicals, reducing oxidative stress and protecting against various types of free radicals, thus preventing neurological disorders^26^. However, information on the link between plant-based dietary patterns and the risk of glioma has gained limited attention.

This study has some priorities over prior publication because previous findings did not consider healthy and unhealthy vegetables separately and they explored the association with cancer only in the context of “vegetarian diets”^27,28^. Earlier studies have classified participants into vegetarians and non-vegetarians and did not take into account the healthy and unhealthy plant-based foods^7,10,29,30^. To our knowledge, no study has evaluated the association of plant-based dietary patterns and risk of glioma. Therefore, this study was conducted to examine the association of Plant-Based Dietary Patterns, including overall plant-based diet (PDI), healthy plant-based diet (hPDI), and unhealthy plant-based diet (uPDI) and risk of glioma in the framework of a case–control study in Iran.

## Methods and materials

### Study design and participants

In this hospital-based case–control study, Iranian specialized centers were regarded as the sampling site. Our study was carried out on 128 glioma patients and 256 controls, between November 2009 and September 2011 in Tehran, Iran. Brain tumors were diagnosed and confirmed by relevant specialists using pathological tests. Eligible subjects based on our inclusion criteria, mentioned below, were enrolled as cases in the current study. Patients with glioma were recruited through a convenience sampling procedure. Controls were selected from other hospital wards (orthopedic and reconstructive surgery) based on their specific inclusion criteria. Cases and controls were matched in terms of age (± 5 years) and sex. Required data were gathered from both cases and controls simultaneously and the same setting. Approval of the study protocol was received from the local Ethics Review Committee at Isfahan University of Medical Sciences, Isfahan, Iran.

### Inclusion and exclusion criteria

The inclusion criteria for recruitment of cases were as follows: (a) pathologically confirmed glioma and utmost one-month interval after glioma diagnosis. (b) Being at the age range of 20–75 y. Controls were included in the study if they were glioma-free and between the ages of 20 and 75. They were matched with cases in terms of sex and age, as mentioned above. Both cases and controls should be enough alert to take part in the study.

We did not include subjects in the study if they were: (a) being in special physiological conditions including breastfeeding or pregnancy (b) having a medical history of some disorders including cancer, neurological, gastrointestinal, hepatic, endocrine, immune, kidney and cardiovascular diseases, (c) not able to follow the protocol of the study, (d) adhering to specific diets different from their routine dietary intakes. Furthermore, a history of chemotherapy or radiation therapy, as well as the use of nitrosamine-enhancing drugs such as nitroglycerin, propranolol, oxytetracycline, and disulfiram, were among our exclusion criteria.

### Dietary intake assessment

To assess habitual dietary intakes (over the past year) of study participants, a Block-format semi-quantitative, valid, and reliable food frequency questionnaire (FFQ) was applied^31^. This questionnaire has consisted of 123 food items, which were commonly used by local people, and a consumption frequency section for all these foods. For each food item in the questionnaire, specific portion size was also given. Participants were requested to specify their frequency consumption (per day, week or month) of each food item during the preceding year, considering each item's portion size. Then, we used the Us Department of Agriculture’s food composition database (modified for Iranian foods)^32^ to compute nutrient intakes for each study participant. To do this, we used Nutritionist IV software (First Databank Division, the Hearst Corporation, San Bruno, CA, USA).

### Assessment of plant-based dietary indices

In our project, we applied Satija et al. method^17^ to construct the required indices; an overall plant-based dietary index (PDI), a healthy plant-based dietary index (hPDI) and an unhealthy plant-based dietary index (uPDI)^20,33^. We used 18 food groups of animal foods, healthy plants, and unhealthy plants in this scoring method. The food groups we used were whole grains, fruits, vegetables, nuts, legumes, vegetable oils, and tea/coffee, which are belonged to the category of healthy plant food groups; refined grains, sugar-sweetened beverages, fruit juices, potatoes, and sweets/desserts which constituted unhealthy plant food groups; and animal fats, dairy, eggs, fish/seafood, meat (poultry and red meat) and miscellaneous animal-based foods. To construct the indices, first, we classified study participants based on quintile cut-points of these 18 food groups and assigned the scores of 1 (the lowest quintile) to 5 (the highest quintile) to each quintile. To calculate PDI, the highest scores were dedicated to plant food groups, while for animal food groups, the lowest scores were allocated. For hPDI, healthy plant food groups were given the highest scores and unhealthy plant food groups and animal food groups were given the lowest scores. The same method was also applied to construct uPDI, where the highest scores were given unhealthy plant food groups and the lowest scores were assigned to healthy plant food groups and animal food groups. Individual’s scores of 18 food groups were added up to finalize these indices. Theoretically, the scores ranged from 18 (as the lowest possible score) to 90 (as the highest possible score). It must be kept in mind that the highest score of all indices represented lower intake of animal foods.

### Assessment of glioma

The pathological test was used to identify glioma using the International Classification of Diseases for Oncology third edition (ICD-O-3) and morphology codes 9380–9481^34^. Glioma patients were accepted into the project after a maximum of one month of disease confirmation.

### Assessment of other variables

A general information pretested questionnaire supplying several sociodemographic variables (age, sex, marriage status, residence place, trade, and education), family history of cancers including glioma, medical history including trauma, hypertension, and allergy, encountering with chemicals over the past ten years, cooking techniques, the experience of hair dyeing, cell phone use duration, encountering with the radiographic x-ray; was administered to reach general information of cases and controls. A short form of the International Physical Activity Questionnaire (IPAQ) was utilized to evaluate each participant's physical activity^35^. The obtained information from the IPAQ was expressed as Metabolic Equivalent per week (METs/week). Weight and height were quantified based on standard methods; body mass index was calculated for each participant. Trained nutritionists collected all these data through a face-to-face method of interview.

### Statistical analysis

Demographic variables, selected risk factors, and dietary intakes between cases and controls were compared using independent samples’ t-test or Chi-square, where appropriate. Then, we categorized all subjects according to tertiles of PDI, hPDI, and uPDI scores. Comparison of continuous and categorical demographic characteristics across tertiles of PDI, hPDI and uPDI scores was done using analysis of variance (ANOVA) and Chi-square, respectively. Dietary intakes of participants across tertiles of PDI, hPDI, and uPDI were compared using analysis of covariance (ANCOVA), which were adjusted for energy intake. The association between PDI, hPDI, and uPDI and the likelihood of having glioma was assessed by binary logistic regression in three different models. In the first model, we adjusted for age (continuous), sex (male/female), and energy intake (continuous). In the second model, additional control was made for physical activity (continues), family history of glioma (yes/no), marital status, high-risk job (farmer/non-farmer), high-risk living area (yes/no), duration of cell phone usage (continues), supplement use (yes/no), history of exposure to the radiographic X-ray (yes/no), history of dental photography (yes/no), history of head trauma (yes/no), smoking status (smoker/non-smoker), exposure to chemicals (yes/no), personal hair dye use (yes/no), frequent use of fried foods (yes/no), and microwave use (yes/no). These confounders were selected based on previous literature^36–39^ as well as significant differences between cases and controls. To obtain the obesity-independent association, we adjusted for BMI (continues) in the last model. The first tertile of these dietary indices was considered as the reference category. To derive the trend of ORs across increasing tertiles of PDI, hPDI and uPDI, these tertiles were considered as an ordinal variable. Data were analyzed using SPSS software (version 22; SPSS Inc, Chicago IL). The P-values of less than 0.05 were considered to characterize significant results.

### Ethical approval

All procedures performed in our study that involved human participants were in accordance with the ethical standards of the institutional and/or national research committee and with the 1964 Helsinki declaration and its later amendments or comparable ethical standards.

### Informed consent

Informed consent was taken from all study participants after acquaintance with the study methodology.

## Results

Demographic characteristics, selected risk factors, and dietary intakes of glioma patients and controls are presented in Table 1. Participants who had glioma were more likely to have high-risk jobs, history of exposure to chemicals, family history of glioma, history of head trauma, history of X-ray radiographic exposure, living in high-risk areas than controls. Cases were also more likely to be frequent fried food consumers, and they had higher intakes of vitamin B12, refined grains, animal fats, and miscellaneous animal-based foods than controls. Contrariwise, the prevalence of cell phone use, history of dental photography, smoking, personal hair dye use, taking supplements, and using microwave prepared foods was higher among controls than cases. They also had a higher consumption of total fats, polyunsaturated fatty acid (PUFA), monounsaturated fatty acid (MUFA), calcium, potassium, fruits, vegetables, nuts, legumes, vegetable oils, tea/coffee, and dairies than cases.

**Table 1:** Demographic characteristics, selected risk factors and dietary intakes of glioma cases and controls.

	Groups		
	Cases (*n* = *128*)	Controls (*n* = *256*)	P*
Age (years)	43.4 ± 14	42.8 ± 13	0.65
BMI (kg m^−2^)	26.2 ± 4.3	26.1 ± 3.8	0.76
Duration of cell phone use (years)	2.8 ± 2.9	3.7 ± 2.5	0.003
Females (%)	41.4	41.8	0.94
Married (%)	78.9	80.1	0.66
High-risk job^a^ (%)	10.2	2.7	0.003
High-risk residential area^b^ (%)	30.5	21.5	0.05
History of exposure to the radiographic X-ray (%)	15.6	7.4	0.01
History of dental photography (%)	46.1	59	0.02
History of head trauma (%)	43.8	28.9	0.004
Current smoker (%)	15.6	25	0.04
Supplement use (%)	7.8	15.6	0.03
Personal hair dye use (%)	21.9	41	< 0.001
Exposure to chemicals (%)	19.5	10.5	0.01
Family history of glioma (%)	19.5	5.5	< 0.001
Family history of cancer (%)	32.8	34	0.82
Frequent fried food intake^c^ (%)	90.6	78.1	0.002
Frequent microwave use^c^ (%)	7.8	19.1	0.004
Frequent canned foods intake^c^ (%)	6.3	5.9	0.88
Physical activity (METs)	34.8 ± 6.3	33.8 ± 5.5	0.12
**Nutrient items**			
Total Energy (Kcal day^−1^)	2580 ± 560	2561 ± 722	0.79
Protein (g day^−1^)	98 ± 22	97 ± 30	0.70
Carbohydrate (g day^−1^)	425 ± 101	412 ± 128	0.31
Fats (g day^−1^)	62 ± 19	66 ± 22	0.05
Dietary fiber (g day^−1^)	23.3 ± 11.2	23.0 ± 14.2	0.82
SFA (g day^−1^)	19.1 ± 7.2	20.7 ± 9.0	0.09
MUFA (g day^−1^)	19.7 ± 6.7	22.0 ± 7.7	0.006
PUFA (g day^−1^)	12.6 ± 3.7	14.2 ± 4.2	0.001
Cholesterol (mg day^−1^)	251 ± 141	235 ± 121	0.24
Folic acid (µg day^−1^)	349 ± 90	382 ± 301	0.23
Vitamin B6 (mg day^−1^)	1.86 ± 0.05	1.97 ± 0.7	0.13
Vitamin B12 (µg day^−1^)	9.65 ± 16.2	5.92 ± 4.5	0.01
Calcium (mg day^−1^)	1019 ± 263	1138 ± 358	0.001
Magnesium (mg day^−1^)	524 ± 133	520 ± 154	0.79
Potassium (mg day^−1^)	4073 ± 782	4363 ± 1422	0.03
**Food groups (g day**^**−1**^**)**			
Whole grains	76.3 ± 87.4	90.9 ± 92.2	0.13
Fruits	311 ± 97	347 ± 123	0.002
Vegetables	246 ± 80	264 ± 79	0.04
Nuts	3.8 ± 3.1	4.9 ± 4.0	0.004
Legumes	40.1 ± 22.5	44.8 ± 19.6	0.03
vegetable oils	7.8 ± 6.2	9.4 ± 5.4	0.01
Tea and coffee	618 ± 299	736 ± 387	0.001
Refined grains	597 ± 199	471 ± 186	< 0.001
Sugar-sweetened beverages	72.9 ± 62.7	61.2 ± 53.3	0.07
Fruit juices	8.1 ± 13.2	7.5 ± 12.3	0.65
Potato	20.7 ± 11.0	20.3 ± 20.0	0.85
Sweet dessert	33.5 ± 17.3	34.3 ± 15.6	0.66
Animal fats	19.4 ± 15.8	12.8 ± 13.4	< 0.001
Dairy	326 ± 116	377 ± 133	< 0.001
Eggs	25.7 ± 17.5	26.6 ± 19.6	0.63
Fish/seafood	13.6 ± 14.6	12.6 ± 11.7	0.53
Meats	63.2 ± 24.3	63.0 ± 33.4	0.93
Miscellaneous animal-based foods	6.9 ± 10.6	4.5 ± 3.8	0.01

All values are mean ± SD or percent.

^a^Farmers were considered as having a high-risk occupation.

^b^Subjects who lived in places nearby electromagnetic fields and cell phone and broadcast antennas in the last 10 years were considered as living in high-risk areas.

^c^Subjects who consumed fried food, microwave and canned foods at least twice per week were considered as frequent users.

*Obtained from independent-samples t-test or Chi-square test, where appropriate.

The general participant characteristics across tertiles of PDI, hPDI, and uPDI are shown in Table 2. Subjects with a higher score of PDI were more likely to have a history of head trauma and exposure to chemicals than those with lower scores. In addition, compared with the participants in the lowest tertile of hPDI, individuals in the highest tertile were more likely to be older, female, married, hair color user and less likely to be current smokers, use cell phone, and consume fried foods. With regard to uPDI, subjects with a greater adherence were more likely to be physically active, have high-risk jobs, and less likely to use cell phone, microwave prepared foods, and take supplements. We found no other significant differences in other general characteristics across tertiles of these dietary indices.

**Table 2:** Demographic characteristics and selected risk factors of the study participants across tertiles of PDI, hPDI, and uPDI scores.

	Tertiles of PDI				Tertiles of hPDI				Tertiles of uPDI			
	T1 < 52	52 < T2 < 57	T3 > 57	P*	T1 < 51	51 < T2 < 56	T3 > 56	P*	T1 < 51	51 < T2 < 57	T3 > 57	P*
Participants (n)	139	112	133		125	132	127		135	127	122	
Age (years)	43.9 ± 14	43.1 ± 14	41.9 ± 12	0.50	39 ± 15	43.4 ± 13^d^	46.4 ± 11	< 0.001	42.1 ± 13	44.4 ± 14	42.4 ± 13	0.35
BMI (kg m^−2^)	26.4 ± 4.2	25.9 ± 4.0	26.1 ± 3.6	0.57	25.9 ± 4.1	26.6 ± 4.2	25.8 ± 3.6	0.24	26.4 ± 3.7	26.1 ± 4.1	25.9 ± 4.0	0.50
Duration of cell phone use (years)	3.4 ± 2.6	3.4 ± 2.8	3.3 ± 2.7	0.97	3.9 ± 2.8	3.4 ± 2.4	2.9 ± 2.7^d^	0.006	4.1 ± 3.1	3.0 ± 2.3^d^	3.0 ± 2.4^d^	0.002
Females (%)	41.7	42.9	40.6	0.93	27.2	35.6	62.2	< 0.001	40.7	41.7	42.6	0.95
Married (%)	77.7	76.8	84.2	0.45	75.2	76.5	87.4	0.02	85.2	71.7	82	0.01
High-risk job^a^ (%)	4.3	7.1	4.5	0.55	4	12	4	0.04	3	5	12	0.02
High-risk residential area^b^ (%)	20.1	24.1	29.3	0.21	25.6	28.8	18.9	0.17	30.4	19.7	23	0.12
History of exposure to the radiographic X-ray (%)	7.2	14.3	9.8	0.18	13.6	6.1	11	0.12	9.6	12.6	8.2	0.50
History of dental photography (%)	57.6	58	48.9	0.25	52.8	55.3	55.9	0.87	59.3	49.6	54.9	0.29
History of head trauma (%)	26.6	34.8	40.6	0.05	36	31.8	33.9	0.77	35.6	33.1	32.8	0.87
Smoking status (yes) (%)	20.9	24.1	21.1	0.79	35.2	19.7	11	< 0.001	20	20.5	25.4	0.52
Supplement use (%)	14.4	13.4	11.3	0.74	13.6	9.1	16.5	0.20	18.5	13.4	6.6	0.02
Personal hair dye use (%)	36	27.7	39.1	0.16	20.8	32.6	50.4	< 0.001	41.5	32.3	29.5	0.10
Exposure to chemicals (%)	7.2	14.3	19.5	0.01	16	15.2	9.4	0.25	14.1	8.7	18	0.09
Family history of glioma (%)	12.2	6.3	11.3	0.26	14.4	9.8	6.3	0.10	8.9	7.1	14.8	0.11
Family history of cancer (%)	30.9	32.1	37.6	0.47	34.4	31.8	34.6	0.86	38.5	36.2	25.4	0.06
Frequent fried food intake^c^ (%)	86.3	81.3	78.9	0.26	91.2	84.1	71.7	< 0.001	78.5	85	83.6	0.34
Frequent microwave use^c^ (%)	15.8	16.1	14.3	0.91	19.2	16.7	10.2	0.12	26.7	10.2	8.2	< 0.001
Frequent canned foods intake^c^ (%)	5	4.5	8.3	0.38	7.2	7.6	3.1	0.25	8.1	7.1	2.5	0.13
Physical activity (METs)	34.1 ± 5.2	34.5 ± 6.2	33.8 ± 5.9	0.68	34.3 ± 6.1	33.5 ± 5.8	34.5 ± 5.4	0.33	34 ± 5.6	33.2 ± 5.5	35.2 ± 6.1^e^	0.03

*PDI* overall plant-based diet index, *hPDI* healthy plant-based diet index, *uPDI* unhealthy plant-based diet index, *MET* metabolic equivalents.

All values are mean ± SD or percent.

^a^Farmers were considered as having a high-risk occupation.

^b^Subjects who lived in places nearby electromagnetic fields and cell phone and broadcast antennas in the last 10 years were considered as living in high-risk areas.

^c^Subjects who consumed fried food, microwave and canned foods at least twice per week were considered as frequent users.

*Obtained from ANOVA with Bonferroni correction or Chi-square test, where appropriate.

^d^Significant compared with T1.

^e^Significant compared with T2.

Dietary intakes of study participants across tertiles of PDI, hPDI, and uPDI scores are presented in Table 3. Individuals in the top tertile of PDI score had higher intakes of total energy, PUFA, magnesium, fruits, vegetables, nuts, legumes, vegetable oils, tea/coffee, sweets/desserts and a lower intake of cholesterol, vitamin B12, animal fats, egg, fish/ seafood, meats, and miscellaneous animal-based foods compared with those in the bottom tertile. In addition, participants with the highest score of hPDI had higher intakes of total fats, MUFA, PUFA, magnesium, whole grains, fruits, vegetables, nuts, legumes, vegetable oils and lower intakes of total energy, cholesterol, vitamin B12, refined grains, sugar-sweetened beverages, fruit juices, potatoes, sweets/desserts animal fats, egg, fish/seafood, meats, and miscellaneous animal-based foods than those with the lowest score. In comparison with subjects in the lowest tertile of uPDI, those in the highest tertile consumed higher amounts of carbohydrates, refined grains, fruit juices, sweets/desserts, animal fats and had lower intakes of total energy, fats, saturated fatty acids (SFA), PUFA, MUFA, cholesterol, vitamin B6, calcium, magnesium, potassium, whole grains, fruits, vegetables, nuts, legumes, vegetable oils, dairy, egg, fish/seafood, and miscellaneous animal-based foods than those with the lowest adherence.

**Table 3:** Dietary intakes of study participants across tertiles of PDI, hPDI, and uPDI scores.

	Tertiles of PDI				Tertiles of hPDI				Tertiles of uPDI			
	T1 < 52	52 < T2 < 57	T3 > 57	P*	T1 < 51	51 < T2 < 56	T3 > 56	P*	T1 < 51	51 < T2 < 57	T3 > 57	P*
**Nutrient items**												
Total Energy (Kcal day^−1^)	2368 ± 54	2467 ± 60	2861 ± 55^ab^	< 0.001	2765 ± 58	2572 ± 57	2368 ± 58^ab^	< 0.001	2850 ± 54	2490 ± 56^a^	2334 ± 57^a^	< 0.001
Protein (g day^−1^)	99 ± 2.5	99 ± 2.8	98 ± 2.7	0.98	99 ± 2.7	101 ± 2.6	97 ± 2.7	0.47	102 ± 2.6	97 ± 2.7	97 ± 2.7	0.34
Carbohydrate (g day^−1^)	414 ± 3.3	418 ± 3.6	416 ± 3.5	0.63	415 ± 3.5	420 ± 3.3	412 ± 3.5	0.28	396 ± 3.2	420 ± 3.2^a^	434 ± 3.3^ab^	< 0.001
Fat (g day^−1^)	65 ± 1.3	64 ± 1.4	65 ± 1.3	0.76	63 ± 1.3	63 ± 1.3	68 ± 1.3^ab^	0.01	73 ± 1.2	63 ± 1.2^a^	56 ± 1.2^ab^	< 0.001
Dietary fiber (g day^−1^)	23 ± 1.1	22 ± 1.2	24 ± 1.1	0.32	21 ± 1.2	24 ± 1.1	23 ± 1.1	0.10	23 ± 1.1	25 ± 1.1	22 ± 1.2	0.20
SFA (g day^−1^)	20 ± 0.6	20 ± 0.7	19 ± 0.6	0.53	20 ± 0.6	19 ± 0.6	21 ± 0.6	0.53	23 ± 0.6	20 ± 0.6^a^	18 ± 0.6^a^	< 0.001
MUFA (g day^−1^)	21 ± 0.5	21 ± 0.6	22 ± 0.5	0.55	20 ± 0.5	21 ± 0.5	22 ± 0.5^a^	0.01	25 ± 0.4	20 ± 0.5^a^	18 ± 0.5^ab^	< 0.001
PUFA (g day^−1^)	13 ± 0.3	13.3 ± 0.3	14.7 ± 0.3^ab^	0.001	12.5 ± 0.3	13.3 ± 0.3	15.3 ± 0.3^ab^	< 0.001	16.3 ± 0.2	12.9 ± 0.3^a^	11.6 ± 0.3^ab^	< 0.001
Cholesterol (g day^−1^)	277 ± 9.3	227 ± 10.4^a^	215 ± 9.8^a^	< 0.001	277 ± 9.8	233 ± 9.5^a^	214 ± 9.9^a^	< 0.001	266 ± 9.8	234 ± 10^a^	222 ± 10.2^a^	0.008
Folic acid (µg day^−1^)	350 ± 21	385 ± 24	380 ± 22	0.47	349 ± 22	391 ± 22	371 ± 23	0.39	386 ± 22	361 ± 23	363 ± 23	0.69
Vitamin B6 (mg day^−1^)	2 ± 0.04	1.9 ± 0.05	1.9 ± 0.05	0.26	1.9 ± 0.05	1.9 ± 0.04	1.9 ± 0.05	0.87	2.1 ± 0.05	1.9 ± 0.05^a^	1.7 ± 0.05^ab^	< 0.001
Vitamin B12 (µg day^−1^)	10.6 ± 0.8	6.1 ± 0.9^a^	4.4 ± 0.8^a^	< 0.001	10.4 ± 0.9	5.9 ± 0.8^a^	5.1 ± 0.9^a^	< 0.001	7.9 ± 0.9	7.0 ±	6.5 ± 0.9	0.60
Calcium (mg day^−1^)	1099 ± 23	1085 ± 26	1108 ± 24	0.80	1092 ± 24	1090 ± 23	1113 ± 24	0.75	1168 ± 23	1094 ± 24	1026 ± 24^a^	< 0.001
Magnesium (mg day^−1^)	508 ± 9	506 ± 10	546 ± 9^ab^	0.005	496 ± 9	527 ± 9	539 ± 9^a^	0.006	546 ± 9	518 ± 9	495 ± 10^a^	0.001
Potassium (mg day^−1^)	4192 ± 85	4182 ± 94	4415 ± 89	0.13	4163 ± 89	4227 ± 86	4409 ± 89	0.14	4600 ± 85	4213 ± 86^a^	3948 ± 88^a^	< 0.001
**Food groups (g day**^**−1**^**)**												
Whole grains	76.4 ± 7.6	93.4 ± 8.3	90 ± 7.9	0.27	63.4 ± 7.9	87.4 ± 7.5	106.8 ± 7.8^a^	0.001	106.3 ± 7.8	72.3 ± 7.8^a^	77.8 ± 8.1^a^	0.006
Fruits	306 ± 8.6	307 ± 9.5	387 ± 9^ab^	< 0.001	305 ± 9.2	318 ± 8.9	381 ± 9.1^ab^	< 0.001	388 ± 8.7	332 ± 8.7^a^	279 ± 9^ab^	< 0.001
Vegetables	233 ± 5.9	247 ± 6.5	293 ± 6.2^ab^	< 0.001	239 ± 6.5	252 ± 6.2	282 ± 6.4^ab^	< 0.001	294 ± 6	257 ± 6^a^	219 ± 6.2^ab^	< 0.001
Nuts	4.0 ± 0.3	4.2 ± 0.3	5.3 ± 0.3^a^	0.01	4.4 ± 0.3	3.8 ± 0.3	5.5 ± 0.3^b^	0.001	6.3 ± 0.3	4.0 ± 0.3^a^	3.1 ± 0.3^ab^	< 0.001
Legumes	37.5 ± 1.6	42.4 ± 1.8	50 ± 1.7^ab^	< 0.001	36.2 ± 1.7	44.2 ± 1.6^a^	49.3 ± 1.7^a^	< 0.001	51.7 ± 1.6	44.3 ± 1.6^a^	32.9 ± 1.6^ab^	< 0.001
vegetable oils	7.6 ± 0.5	8.4 ± 0.5	10.5 ± 0.5^ab^	< 0.001	7.1 ± 0.5	8.3 ± 0.5	11.2 ± 0.5^ab^	< 0.001	11.4 ± 0.5	8.4 ± 0.5^a^	6.5 ± 0.5^ab^	< 0.001
Tea and coffee	605 ± 31	714 ± 34^a^	777 ± 32^a^	0.001	686 ± 33	716 ± 32	687 ± 33	0.75	724 ± 32	673 ± 32	691 ± 34	0.54
Refined grains	519 ± 14	503 ± 16	516 ± 15	0.74	552 ± 15	548 ± 14	438 ± 14^ab^	< 0.001	436 ± 14	523 ± 14^a^	588 ± 15^ab^	< 0.001
Sugar-sweetened beverages	68.3 ± 4.6	66.2 ± 5.1	61 ± 4.8	0.55	89.7 ± 4.6	63.9 ± 4.4	42.3 ± 4.5	< 0.001	66.4 ± 4.8	65.1 ± 4.8	63.8 ± 5.0	0.93
Fruit juices	6.0 ± 1.0	9.5 ± 1.2	7.9 ± 1.3	0.09	12.2 ± 1.1	6.4 ± 1.0^a^	4.5 ± 1.1^ab^	< 0.001	4.6 ± 1.1	9.0 ± 1.1^a^	9.7 ± 1.1^a^	0.003
Potato	19.9 ± 1.4	18.3 ± 1.5	22.4 ± 1.4	0.24	24.6 ± 1.4	17.9 ± 1.4^a^	19 ± 1.4^a^	0.003	18.6 ± 1.4	20.9 ± 1.4	21.9 ± 1.5	0.29
Sweet/dessert	31.1 ± 1.3	34.5 ± 1.5	36.6 ± 1.4^a^	0.02	37.9 ± 1.3	35.8 ± 1.3	28.3 ± 1.3^ab^	< 0.001	30.3 ± 1.3	34.5 ± 1.3	37.6 ± 1.4^a^	0.002
Animal fats	18.6 ± 1.2	15.1 ± 1.3	11.1 ± 1.3^a^	< 0.001	20.5 ± 1.2	15.9 ± 1.2^a^	8.6 ± 1.2^ab^	< 0.001	11.8 ± 1.3	15.4 ± 1.3	18.1 ± .3^a^	0.004
Dairy	376 ± 10	349 ± 11	353 ± 11	0.17	372 ± 11	346 ± 10	362 ± 11	0.22	402 ± 10	360 ± 10^a^	313 ± 11^ab^	< 0.001
Eggs	29.7 ± 1.4	24.2 ± 1.6^a^	24.5 ± 1.5^a^	0.01	31.3 ± 1.5	26.3 ± 1.4	21.4 ± 1.5^a^	< 0.001	30.7 ± 1.5	24.7 ± 1.5^a^	23.1 ± 1.5^a^	0.002
Fish/seafood	17.1 ± 1.0	11.6 ± 1.1^a^	9.7 ± 1.1^a^	< 0.001	15.2 ± 1.1	12.5 ± 1.0	11.3 ± 1.1^a^	0.04	15.7 ± 1.0	14 ± 1.0	8.9 ± 1.1^ab^	< 0.001
Meats	67.2 ± 2.2	63.7 ± 2.5	58.1 ± 2.3^a^	0.02	70.8 ± 2.3	62.1 ± 2.2^a^	56.5 ± 2.3^a^	< 0.001	67.5 ± 2.3	61.6 ± 2.3	59.6 ± 2.4	0.06
Miscellaneous animal-based foods	7.5 ± 0.5	4.3 ± 0.6^a^	3.8 ± 0.6^a^	< 0.001	7.9 ± 0.6	4.6 ± 0.5^a^	3.4 ± 0.6^a^	< 0.001	6.4 ± 0.6	5.3 ± 0.6	4.1 ± 0.6^a^	0.03

*PDI* overall plant-based diet index, *hPDI* healthy plant-based diet index, *uPDI* unhealthy plant-based diet index, *SFA* saturated fatty acid, *PUFA* polyunsaturated fatty acid, *MUFA* monounsaturated fatty acid.

Data are presented as mean ± SE.

*All values, except energy intake, were adjusted for energy intake using ANCOVA with Bonferroni correction.

^a^Significant compared with T1.

^b^Significant compared with T2.

**Figure 1:**
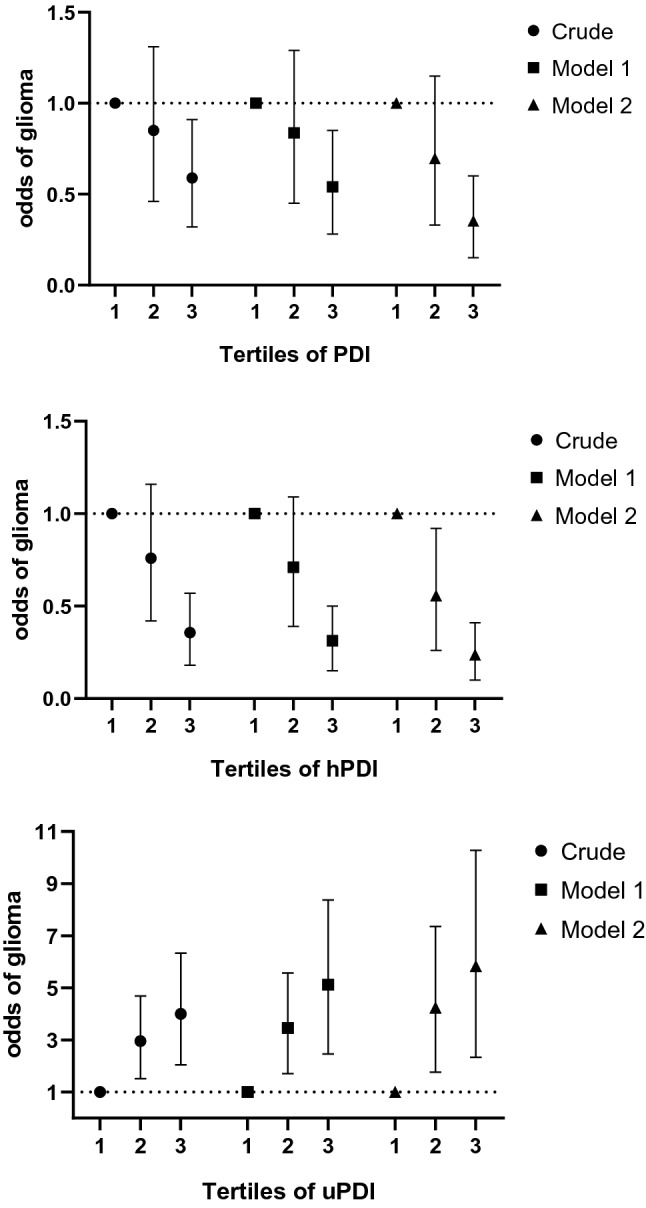
Crude and multivariable-adjusted odds ratios and 95% CIs of glioma by tertiles of different plant-based diet index. Model 1: adjusted for age, sex, and energy intake. Model 2: further adjustments were made for physical activity, family history of glioma, marital status, high-risk job, high-risk living area, duration of cell phone usage, supplement use, history of exposure to the radiographic X-ray, history of dental photography, history of head trauma, smoking status, exposure to chemicals, personal hair dye use, frequent use of fried food, and microwave.

Crude and multivariable-adjusted odds ratios and 95% confidence intervals (CIs) for glioma across tertiles of PDI, hPDI, and uPDI are outlined in Fig. 1. After adjustment for age, sex, energy intake, and potential confounders, there was an inverse significant association between PDI (OR: 0.31, 95% CI: 0.15–0.60, *P*-trend = 0.001) and hPDI (OR: 0.28, 95% CI: 0.13–0.6, *P*-trend = 0.001) and odds of glioma. When multiple variables were included in the second model, the effect measure increased, for which the greater impact was due to the inclusion of history of head trauma, smoking, residential area, and chemical exposure. In other words, when these variables were excluded, the OR for PDI was 0.45 (95% CI: 0.25–0.82) and for hPDI was 0.28 (95% CI: 0.14–0.55). Furthermore, when BMI was taken into account, the association did not change much for PDI (OR: 0.31, 95% CI: 0.16–0.62, *P*-trend = 0.001) and hPDI (OR: 0.21, 95% CI: 0.10–0.42, *P*-trend < 0.001). A significant positive association was seen between uPDI and glioma in all models. Subjects in the highest tertile of uPDI had 4.89 times higher chance of glioma than those in the lowest tertile (OR: 4.89, 95% CI: 2.33–10.28, *P*-trend < 0.001). When potential confounders were considered in the second model, the effect measure strengthened, and the greater impact was due to adjustment for history of head trauma, smoking, residential area, and chemical exposure. After removing these variables from the model, the OR for uPDI was 3.32 (95% CI: 1.68–6.58). Additional adjustment for BMI had no effect on the observed association (OR: 4.78, 95% CI: 2.27–10.08, *P*-trend < 0.001).

## Discussion

In this hospital-based case–control study of a sample of Iranian adults, we found that greater adherence to PDI and hPDI had a significant inverse association with a chance of glioma, while higher adherence to uPDI was directly associated with the likelihood of glioma. These associations were independent of multiple potential confounders, including energy intake, several environmental parameters, and BMI. To the best of our knowledge, this is the first study investigating the association between plant-based dietary patterns and risk of glioma.

Glioma is a prevalent type of brain tumor that is considered to be the most common tumor in the central nervous system^40^. Despite recent developments in treatment, nearly two-thirds of patients do not survive more than two years after diagnosis^41^. This highlights the primary prevention of glioma. Epidemiological studies reported various unchangeable factors such as age, ethnicity, gender, hormonal status, genetic, and several modifiable factors including exposure to radiation, cell phone usage, lifestyle, and diet are associated with the development of glioma^42^. In this regard, there is numerous evidence to support that changing diet can remarkably reduce the incidence of various cancers, especially glioma^43,44^. It has been well established that the whole diet compared with individual foods or nutrients might be better to represent the overall quality of the diet and help to predict the link between diet and disease^45,46^. Earlier investigations have mainly categorized foods into two main groups: animal and plant foods, regardless of no differentiation between healthy and unhealthy plant foods^47^. However, several plant-based foods, such as refined grains, potatoes, and sugar-sweetened beverages, have been associated with chronic diseases^10,18,19^. Therefore, in the current study in addition to evaluating the effect of an overall plant-based diet, we also separately evaluated the effect of healthy and unhealthy plant foods.

The present study results indicated that greater scores to PDI and hPDI were associated with lower odds of glioma. In line with our findings, several recent studies have shown the protective effect of plant-based diets on the risk of cancers. Results of the French NutriNet-Santé prospective cohort study, performed among 42,544 participants, indicated that the overall incidence of cancer among those with a higher plant-based dietary score was lower than those with the lowest scores. When particular cancers were assessed, they found an inverse association only for digestive and lung cancers. However, they no longer examined the effect of “healthy” or “unhealthy” plant-based diets^48^. In a meta-analysis of 10 cohort studies, an inverse association was reported between a special vegetarian diet and the incidence of total cancer^49^. In another prospective study, including 7216 participants, and a mean follow-up of 4.8 years, higher adherence to a priori-defined plant-based diet was inversely associated with mortality from all causes. When they examined cancer mortality, there was no statistically significant relationship, which can be attributed to the incidence of only 130 deaths from cancer, and the absence of adequate statistical power to find the real associations^50^. In addition, Xiao et al., in a recent meta-analysis of observational studies, suggested that the western dietary pattern that is characterized by high intakes of animal foods was associated with an increased risk of breast cancer^51^. The different nature of glioma from other cancers as well as the association between this new plant-based dietary index and risk of glioma had never been explored. Thus it was not possible to directly compare our findings with similar epidemiological studies.

Several physiological mechanisms from experimental studies supported the observed favorable effect of adherence to the plant-based diet and risk of glioma. Plant-based dietary patterns contain a high quantity of whole grains, vegetables, fruits, legumes, and nuts, and these foods are rich in antioxidants, polyphenols, vitamins, and minerals^52,53^. Previous clinical trials and observational studies have clearly shown that these components individually or jointly improve oxidative stress and inflammation, producing reactive oxygen species, and taking part in glioma pathogenesis^54,55^. In addition, higher intakes of fiber and phytochemicals reduce inflammation, circulating estrogens and androgens hormones, insulin resistance, and Insulin-like Growth Factor-1 concentration play a key role in cancer prevention^56,57^. On the other hand, lower intakes of animal products may be another possible mechanism. These foods contain a large amount of heme iron, which may involve initiating and promoting carcinogens through several mechanisms, including the production of free radicals, N-nitroso compounds, or lipid peroxidation^58–60^. A newly understood mechanism that has been considered in recent years is related to the gut microbiome^61^. In brief, a healthy plant-based diet may reduce the risk of cancer by altering the gut microbial environment, which promotes the metabolism of fiber and polyphenols while inhibiting the metabolism of choline, bile acids, amino acids, and L-carnitine^62^.

We also found that higher scores of uPDI were associated with an increased risk of glioma. This dietary index was characterized by high quantities of less healthy plant foods. Based on the magnitude of the estimates, the association with uPDI appears to be the strongest result. These foods have a higher content of carbohydrate, sodium, added sugar, and are poor in healthy fats, fiber, minerals, and vitamins^17^. Prior studies have demonstrated that frequent consumption of sugar-sweetened beverages (SSBs) or refined grains was associated with declined cognitive function^63^, increased risk of obesity^64^, and glucose metabolism disorders^65^, all of which are well-known factors for the development of brain tumors. These foods have a high glycemic index that leads to a rapid increase in insulin^66^ and IGF-1 production^67^; all of which have been passed the blood–brain barrier and promote the development of glioma^68^.

Several strengths of our project are worth noting. To our knowledge, this is the first study investigating the association between plant-based dietary indices and risk of glioma. Besides, adjusting for a wide range of potential confounders in the analysis was done to reach an independent link between plant-based dietary patterns and glioma odds. In addition, due to selecting cases from newly-diagnosed glioma patients, the possibility of changed dietary habits after glioma diagnosis can be ruled out. Finally, most previous studies have modeled plant-based diets as a binary variable considering vegetarians vs. non-vegetarians, while we used these dietary factors on a continuum with more or fewer plant-based foods that enabled us to continuously describe the adherence to plant-based dietary patterns. However, some limitations need to be highlighted. Due to the nature of the case–control design, the inherent possibility of selection and recall bias was not conceivable. Also, misclassification of study participants is possible due to the use of FFQ. Despite controlling for wide range confounders, the possibility of residual confounding cannot be ignored. Given the limited sample size as well as differences in the Middle Eastern population's ethnicity and dietary patterns with Western nations, extrapolating of our findings to the other populations should be done cautiously. In this project, adherence to different types of plant-based diets was assessed using sample-based scoring of plant-based diets since there is no clear thresholds for absolute levels of intake of plant and animal foods. Finally, we did not collect data on major histological types of disease in the current study.

In conclusion, this hospital-based case–control study indicated that adherence to PDI and hPDI was inversely associated with odds of glioma; conversely, higher scores of uPDI was related to an increased chance of glioma. Our findings support current recommendations to follow diets rich in healthy plant foods, with lower consumption of less healthy plant and animal foods. Future prospective and interventional studies are essential to examine our understanding of the effect of different plant-based diets on glioma.
